# Low Cerebrospinal Fluid Levels of Melanotransferrin Are Associated With Conversion of Mild Cognitively Impaired Subjects to Alzheimer’s Disease

**DOI:** 10.3389/fnins.2019.00181

**Published:** 2019-03-08

**Authors:** Azhaar Ashraf, Jose Andres Alepuz Guillen, Manal Aljuhani, Chantal Hubens, Po-Wah So

**Affiliations:** Department of Neuroimaging, Institute of Psychiatry, Psychology and Neuroscience, King’s College London, London, United Kingdom

**Keywords:** Alzheimer’s disease, CSF, iron, MCI, melanotransferrin, microglia

## Abstract

The disruption of iron metabolism and iron transport proteins have been implicated in the pathogenesis of Alzheimer’s disease (AD). Serum melanotransferrin (MTf), a transferrin homolog capable of reversibly binding iron, has been proposed as a biochemical marker of AD. MTf has also been shown to be elevated in iron-rich reactive microglia near amyloid plaques in AD. We examined the association of CSF MTf to hippocampal volumes and cognitive tests in 86 cognitively normal, 135 mild cognitive impairment (MCI) and 66 AD subjects. CSF was collected at baseline for MTf, Aβ, total-tau and phosphorylated-tau measurements. Serial cognitive testing with ADAS-Cog13, Rey’s auditory visual learning test (RAVLT), mini-mental state examination (MMSE) were performed alongside hippocampal MRI volumetric analysis for up to 10 years after baseline measurements. High levels of baseline CSF MTf were positively associated with baseline hippocampal volume (*R*^2^ = 22%, β = 0.202, and *p* = 0.017) and RAVLT scores (*R*^2^ = 7.30%, β = -0.178, and *p* = 0.043) and negatively correlated to ADAS-Cog13 (*R*^2^ = 17.3%, β = 0.247, and *p* = 0.003) scores in MCI subjects. Interestingly, MCI subjects that converted to AD demonstrated significantly lower levels of CSF MTf (*p* = 0.020) compared to MCI non-converters at baseline. We suggest the diminished CSF MTf observed in MCI-converters to AD may arise from impaired transport of MTf from blood into the brain tissue/CSF and/or increased MTf export from the CSF into the blood arising from attenuated competition with reduced levels of CSF Aβ. Further investigations are required to determine the source of CSF MTf and how brain MTf is regulated by cellular barriers, Aβ and activated microglia that surround plaques in AD pathophysiology. In conclusion, low CSF MTf may identify those MCI individuals at risk of converting to AD.

## Introduction

A growing body of evidence implicates iron metabolism as a contributing factor to oxidative stress and neurodegeneration in Alzheimer’s disease (AD) (Meadowcroft et al., 2015; Ashraf et al., 2018). The transition metal, iron, is crucial in essential processes including DNA synthesis, myelin synthesis, neurotransmitter synthesis and metabolism in the central nervous system (Ward et al., 2014). Iron has been shown to associate with insoluble amyloid plaques (Telling et al., 2017) and neurofibrillary tangles (Yamamoto et al., 2002), characteristic hallmarks of AD. Iron produces reactive oxygen species (ROS) via the Fenton reaction, damaging macromolecules such as lipids, proteins and nucleic acids (Smith et al., 1997; Ward et al., 2014; Ashraf et al., 2018). Deferoxamine, an iron chelator, demonstrated substantial improvement in cognitive performance in AD subjects (Crapper McLachlan et al., 1991).

Melanotransferrin (MTf) or p97 belongs to the transferrin superfamily and binds to a single ferric iron with high affinity (Baker et al., 1992). It has been demonstrated to exist as a plasma membrane glycosyl phosphatidylinositol (GPI) anchored protein (Alemany et al., 1993; Kennard et al., 1995) or a soluble and actively secreted protein (Food et al., 1994; Desrosiers et al., 2003). The physiological function of both forms of MTf remain to be established. MTf is expressed by brain capillary endothelium in cognitively normal (CN) individuals, but also shown to be specifically localized in the reactive microglia associated with senile plaques in AD brains (Jefferies et al., 1996; Rothenberger et al., 1996; Yamada et al., 1999). MTf levels have been demonstrated to be increased in the serum of AD subjects compared to healthy controls, and significantly increased in cerebrospinal fluid (CSF) of AD subjects compared to individuals suffering from other CNS diseases (Kennard et al., 1996; Feldman et al., 2001; Kim et al., 2001), highlighting the potential of MTf as a possible AD biomarker. Desrosiers et al. (2003) demonstrated no differences in levels of serum MTf between AD and control subjects. However, the study was not only statistically underpowered with a small sample size, the ages and sex of subjects were not reported, and also suffered from methodological concerns. The latter include non-optimal preparation and storage of samples; lack of a calibration curve for absolute quantification and a positive control for western blot analysis (as described in Kennard et al., 1996). In contrast, other groups independently validated the use of serum MTf as a potential biomarker of AD in statistically well-powered double-blind studies (Feldman et al., 2001; Kim et al., 2001). The aim of the present study was to determine the association of baseline CSF MTf with AD biomarkers, cognitive and neuroimaging measures using the AD Neuroimaging Initiative (ADNI) cohort. We hypothesized that increased CSF MTf levels will be associated with cognitive impairment in the ADNI cohort.

## Materials and Methods

A total of 287 Alzheimer’s Disease Neuroimaging Initiative (ADNI) subjects comprising of 86 CN, 135 mild cognitive impaired (MCI) and 66 AD subjects were included in the present study^1^. Of the MCI subjects, 85 converted to AD (MCI-c), while the remaining 50 MCI-nc did not in a period of 10 years, with most continuing to satisfy the criteria for MCI with the exception of four, who became CN. CN subjects had MMSE scores of ≥25 and no history of significant cognitive or physical impairments. MCI subjects had a MMSE score of ≥24, a memory complaint but preservation of cognitive and functional performance. AD cases included had an MMSE score of ≥20 and met the NINCDS/ADRDA criteria for probable AD. Detailed inclusion/exclusion criteria are available on the ADNI website^2^. Subjects included in the study underwent lumbar puncture and blood collection at baseline; and serial cognitive testing – AD Assessment Scale-Cognitive Subscale (ADAS-Cog13) and Rey’s auditory visual learning test (RAVLT) alongside magnetic resonance imaging (MRI)-assessment of hippocampal volume. Although not all subjects had [^18^F]FDG-PET (^18^F-fluorodeoxy-glucose positron emission tomography), the data was still included to provide information about synaptic glucose metabolism. ADNI uses serial clinical and neuropsychological assessments (MRI), PET, and baseline CSF biomarkers, in combination to monitor progression of MCI subjects to AD. ADNI was approved by the institutional review board and ethics committees of participating institutions, and written informed consent was obtained from all participants or their next of kin.

### CSF Analysis

Participants underwent CSF sampling as described fully on the ADNI website^2^. Briefly, a small sample of CSF was collected from the lower spine by lumbar puncture in the morning following an overnight fasting. Samples were frozen on dry ice within an hour of collection, and consequently shipped on dry ice to the ADNI Biomarker Core laboratory (University of Pennsylvania Medical Center). Aliquots of 500 μl were prepared after an hour of thawing at ambient temperature and gently mixed. The aliquots were kept at -80°C prior to analysis.

Cerebrospinal fluid Aβ_1-42_, total-tau (ttau) and phosphorylated-tau (181p, ptau) were measured using the multiplex xMAP Luminex platform (Shaw et al., 2011). A multiplexed mass spectrometry (MS)-based assay using multiple reaction monoring (MRM) was used to detect CSF levels of MTf and developed by Caprion Proteomics in collaboration with the ADNI Biomarker Consortium Project team. The technology, quality control and validation of the MRM platform is fully described in the “Use of Targeted Mass Spectrometry Proteomic Strategies to Identify CSF-Based Biomarkers in Alzheimer’s Disease Data Primer”^3^. Briefly, but fully described in the primer and elsewhere (Spellman et al., 2015), CSF (100 μl) was depleted of plasma proteins using a Multiple Affinity Removal System (MARS-14) column and digested with trypsin (1:25 protease:protein ratio). Following lyophilization, samples were desalted and reconstituted with five internal standard peptides and analyzed by LC/MRM-MS on a 5500 QTRAP LC-MS/MS system: Q1 isolates the characteristic MTf (trypsin-digested) peptide ion (TRFM_ADTDGGLIFR) which then undergoes collision-induced-dissociation in Q2 to produce a characteristic fragment ion measured in Q3. The signal of the fragment ion was monitored over the chromatographic elution time and used for quantification. The peptide, TRFM_ADTDGGLIFR, had been previously synthesized and used for method development prior to analysis. Also, note that absolute quantification by an external standard in a different matrix (and fully described in the data primer mentioned above) is only an approximation. While absolute quantification is possible with spiking of known amounts of stable isotope-labeled peptides into samples, this was not done, however, data comparisons between subjects remain valid.

### Structural MRI Volumes

Subjects underwent structural T1-weighted MRI at 1.5T using a sagittal 3D-volumetric magnetization prepared rapid gradient echo (MP-RAGE) sequence (Jack et al., 2008). Briefly, the acquisition parameters were: repetition time (for the inversion pulses), 2400–3000 ms; echo time, 4 ms; inversion time, 1000 ms and 8° flip angle. The field of view was 240 mm × 240 mm, matrix size 192 mm × 192, and 1.2 mm thick 160–208 slices collected covering the whole brain to give a nominal resolution of 0.94 mm × 0.94 mm × 1.2 mm. Hippocampal volumes were obtained using FreeSurfer (version 4.1.0) and fully detailed elsewhere (Fischl et al., 2002, 2004). In brief, motion correction, affline transformation to Talairach image space, intensity inhomogeneity, and removal of non-brain tissues were performed. Following intensity normalization and non-linear warping of the atlas brain image to the subject image, the resultant warped atlas brain image underwent atlas-based tissue segmentation to label various brain regions including the hippocampus. Hippocampal volume was calculated by multiplying the number of voxels by the voxel volume. MRI was performed at baseline, 6 months, 1 year, then yearly for 10 years.

### [^18^F] Fluorodeoxyglucose ([^18^F]FDG-PET)

[^18^F]FDG-PET scans were acquired on multiple scanners with various resolutions, e.g., voxel dimensions of 2.0 mm × 2.0 mm × 2.0 mm with image size, 128 × 128 × 63, at 6 months, 1, 1.5, and 2 years (Jagust et al., 2010). The scans were acquired as 6 × 5-min images, from 30 min after injection of 185 MBq (5 mCi) of [^18^F]-FDG (for full details^4^). Each image was registered to the first image to produce a dynamic image set which was then averaged to yield a single 30-min PET image. For comparison between subjects, each baseline average PET image was reoriented along the anterior-posterior commissure line and resliced to a 1.5 mm isotropic voxel space and smoothed using a standard 14 mm full-width half-maximum kernel to produce images of a uniform resolution. Each PET image was spatially normalized to Montreal Neurological Institute brain space and the mean hippocampal FDG uptake (normalized to pons uptake) measured^5^.

### Neuropsychological Assessments

All subjects underwent detailed neuropsychological testing including ADAS-Cog13 and RAVLT. ADAS-Cog13 is a 13-item scale used for assessing learning, memory, language production and comprehension, constructional and ideational praxis, orientation, has number cancelation and delayed free recall tasks. The word recall test was administered first, and the word recognition task given at the end with other cognitive tasks given in between. The two-word memory tasks were separated so that the risk of individuals confusing words from the two tasks was minimized. Objective testing was followed by subjective clinical ratings of language ability and aptitude of the participant to remember test instructions. The test is scored in terms of errors and range from 0 to 70, with higher scores indicative of poor performance^6^.

The RAVLT tests episodic verbal memory by assessing an individual’s ability to acquire a list of 15 unrelated words (all nouns) over five trials. The words are presented orally to the subject at a rate of one word per second and immediate free recall of words is elicited. The number of correctly recalled words are recorded on each trial. Following a 30-min delay filled with unrelated testing (distractor list), the subject is required to repeat the original list of 15 words. Finally, a yes/no recognition trial is administered which consists of the original 15-words and 15 randomly interspersed distractor words. The number of target “hits” as well as false positive responses are recorded. The sum of scores from the first five trials was used to compute the RAVLT score. Cognitively intact individuals attain a higher score than individuals exhibiting cognitive impairment^6^.

### Statistical Analysis

ANCOVA models assessed differences in CSF levels of MTf, Aβ, tau, neuropsychological tests and neuroimaging measures across diagnostic groups, with age, sex and APOE𝜀4 status as covariates. Since age, sex and genetic status have been known to affect the dependent variables under study, ANCOVA was chosen to adjust for the variance attributed to these factors (covariates), to understand the effect of disease on the dependent variables in question. The CSF Aβ and MTf were normally distributed, while ttau and ptau were natural log-transformed to ensure normality. For regression models, we tested the conditions necessary to satisfy assumptions by checking for collinearity, normal distribution of residuals, maintenance of homoscedasticity and normality of error terms. All models satisfied these conditions. Associations between baseline cognitive scores and neuroimaging measures as well as percentage longitudinal change, i.e., (follow time measure–baseline measure)/baseline measure × 100%, with baseline MTf were performed using linear regression. Since follow-up times were different between subjects, follow-up time was included as a covariate. We then used two-tailed *T*-test to determine differences in baseline MTf levels between MCI converters (MCI-c, *n* = 85) and non-converters (MCI-nc, *n* = 50) to AD. A *p*-value of ≤0.05 was considered significant. All analysis was performed using SPSS IBM version 22.0 and GraphPad Prism 7.0 (GraphPad Inc., San Diego, CA, United States) was used to produce figures.

## Results

The demographics of individuals based on diagnosis are shown in Table 1, and for MCI-nc and MCI-c in Table 2, while Supplementary Table 1 document the follow-up time for CN, MCI, and AD subjects. Levels of CSF MTf were not significantly different between CN, MCI, and AD subjects (Table 1). However, multiple regression modeling of established AD biomarkers and MTf in the total cohort showed higher levels of CSF MTf were positively associated with hippocampal volume (*R*^2^ = 38.0, β = 0.169, *p* = 0.001; Table 3 and Supplementary Figure 1) and percentage longitudinal change in RAVLT scores (*R*^2^ = 20.6, β = 0.127, *p* = 0.025; Table 3 and Supplementary Figure 2).

**Table 1:** Demographics of subjects stratified by diagnosis: cognitively normal (CN), mild cognitive impairment (MCI), and Alzheimer’s disease (AD).

	CN	MCI	AD	*p*-value
*n*	86	135	66	NA
Age (years)	75.70 (5.54)	74.69 (7.35)	74.98 (7.57)	0.448
Female, *n* (%)	42 (48.83)	44 (32.59)	29 (43.94)	***0.043***
Ethnicity:				NA
White Hispanic	1	3	0	
White non-Hispanic	77	128	66	
Black non-Hispanic	8	2	0	
Asian non-Hispanic	0	3	0	
Education (years)	15.56 (2.97)	16 (2.97)	15.11 (2.96)	0.133
APOE𝜀4 +ve, *n* (%)	21 (24.42)	71 (52.59)	47 (71.21)	***1.26 ×10*^-^*^8^***
CSF MTf (a.u.)	8.69 (0.46)	8.71 (0.45)	8.68 (0.42)	0.733
CSF Aβ (pg/ml)	257.42 (20.39)	162.06 (51.32)	145.72 (44.96)	***8.61 ×10*^-^*^8^***
CSF ttau (pg/ml)	62.58 (25.35)	99.30 (50.38)	134.64(68.46)	***1.06 ×10*^-^*^7^***
CSF ptau (pg/ml)	21.42 (9.62)	34.42 (14.50)	44.91 (24.04)	***4.60 ×10*^-^*^7^***
MMSE score	29.06 (1.03)	26.92 (1.74)	23.52 (1.85)	***1.76 ×10*^-^*^48^***
ADAS-Cog13 score	10.19 (4.38)	18.85 (6.65)	30.87 (9.53)	***1.44 ×10*^-^*^38^***
RAVLT score	42.16 (7.88)	30.12 (8.44)	21.76 (6.82)	***1.32 ×10*^-^*^34^***
Hippocampal Volume (mm^3^)	7153.11 (772.89)	6346.59 (1142.55)	5749.67 (1093.18)	***7.82 ×10*^-^*^16^***
Hippocampal FDG (a.u.)	1.31 (0.15)	1.20 (0.13)	1.07 (0.13)	***1.28 ×10*^-^*^7^***


*Data is presented as mean ± standard deviation. P-values are presented for ANCOVA models of CSF proteins, hippocampal volume and FDG, cognitive measures and AD biomarkers, with adjustment for age, sex and APOE𝜀4 status, with significant *p*-values denoted by bold italics (a.u., arbitrary units).*

**Table 2:** Demographics of MCI subjects based on their conversion status: MCI non-converters (MCI-nc) and converters (MCI-c) to AD.

	MCI-nc	MCI-c	*p*-value
N	50	85	NA
Age (years)	75.10 (6.92)	74.46 (7.62)	0.618
Female, *n* (%)	16 (32)	28 (32.94)	0.911
Education (years)	16.26 (2.86)	15.85 (3.03)	0.430
APOE𝜀4 +ve, *n* (%)	22 (44)	49 (57.65)	0.129
CSF MTf (a.u.)	8.82 (0.44)	8.64 (0.44)	***0.020***
CSF Aβ (pg/ml)	180.17 (54.95)	149.76 (43.29)	***0.001***
CSF ttau (pg/ml)	96.28 (52.09)	106.99 (51.32)	0.115
CSF ptau (pg/ml)	31.94 (15.24)	37.20 (14.80)	***0.033***
MMSE score	27.56 (1.59)	26.54 (1.72)	***0.001***
ADAS-Cog13 score	16.51 (5.83)	20.50 (5.84)	***2.28 ×10*^-^*^4^***
RAVLT score	33.44 (9.54)	27.88 (7.02)	***0.001***
Hippocampal Volume (mm^3^)	6662.65 (1050.19)	5940.97 (1010.71)	***2.95 ×10*^-^*^4^***
Hippocampal FDG (a.u.)	1.25 (0.14)	1.17 (0.11)	***0.014***


*Data is presented as mean ± standard deviation. *P*-values presented for two-tailed *T*-test of CSF proteins, hippocampal volume and FDG, cognitive measures and AD biomarkers, with significant *p*-values denoted in bold italics (a.u., arbitrary units).*

**Table 3:** Modeling the association of CSF Aβ, total tau (ttau), phosphorylated tau (ptau), and melanotransferrin (MTf) with neuroimaging measures of the hippocampus and cognitive scores in the total cohort (Supplementary Figures 1, 2).

	Aβ		Ttau		Ptau		MTf		Adjusted R^2^ (%)
	β	*p*	β	*p*	β	*p*	β	*p*	
**Neuroimaging measures**					
Volume	0.027	0.654	-0.028	0.739	-0.138	0.107	0.169	***0.001***	38.0
Volume % change	0.265	***6.1* × *10*^-^*^5^***	0.079	0.390	-0.200	***0.033***	0.066	0.227	34.7
FDG	0.023	0.783	0.208	0.107	-0.309	***0.014***	0.115	0.129	29.4
FDG % change	0.165	0.058	-0.071	0.588	-0.088	0.495	0.087	0.274	17.4
**Cognitive measures**									
MMSE	0.041	0.389	0.027	0.684	-0.042	0.533	0.012	0.769	59.4
MMSE % change	0.298	***8.0 ×10*^-^*^6^***	-0.033	0.724	-0.043	0.645	0.080	0.151	23.1
ADAS-Cog13	-0.121	***0.011***	0.043	0.520	-0.010	0.888	-0.076	0.059	59.0
ADAS-Cog13 % change	-0.193	***0.005***	-0.037	0.697	0.125	0.203	-0.017	0.770	15.9
RAVLT	0.067	0.211	-0.087	0.250	0.020	0.798	0.025	0.574	48.5
RAVLT % change	0.244	***3.0 ×10*^-^*^4^***	0.084	0.370	-0.099	0.297	0.127	***0.025***	20.6


*Age, sex and diagnosis were included as covariates. The % change represents the longitudinal change in neuroimaging/cognitive measures from baseline to the follow-up period for each respective patient. Since the follow-up time points were different between subjects, we included the follow-up time as a covariate. Data is presented as mean ± standard deviation. The standardized coefficient (β) with adjusted R^2^ and *p*-values are stated, with significant *p*-values in bold italics.*

Multiple regression modeling was repeated to determine the associations between established AD biomarkers, cognitive scores and MTf based on diagnosis (Tables 4, 5 and Supplementary Figures 3, 4). The regression model demonstrated higher levels of CSF MTf were positively associated with hippocampal volume in CN (*R*^2^ = 24.8, β = 0.311, *p* = 0.005; Table 4 and Supplementary Figure 3A) and MCI individuals (*R*^2^ = 21.6, β = 0.206, *p* = 0.016; Table 4 and Supplementary Figure 3A). Although CSF MTf was not associated with hippocampal volume in AD, MTf were associated with longitudinal hippocampal volume change (*R*^2^ = 43.8, β = 0.288, *p* = 0.036; Table 4 and Supplementary Figure 3A). Also, CSF MTf was positively correlated to baseline glucose metabolism (*R*^2^ = 17.7, β = 0.426, *p* = 0.019; Table 4 and Supplementary Figure 3B) in AD subjects. The only associations between CSF MTf and cognitive scores were observed in MCI, with MTf negatively associated with ADAS-Cog13 scores (*R*^2^ = 7.00, β = -0.172, *p* = 0.050; Table 5 and Supplementary Figure 4C) and positively associated with longitudinal change in RAVLT scores (*R*^2^ = 17.6, β = 0.248, *p* = 0.003; Table 5 and Supplementary Figure 4C).

**Table 4:** Modeling the association of CSF Aβ, total tau (ttau), phosphorylated tau (ptau), and melanotransferrin (MTf) with neuroimaging measures of the hippocampus in cognitively normal (CN), mild cognitive impairment (MCI) or Alzheimer’s disease (AD) (Supplementary Figure 3).

	Aβ		Ttau		Ptau		MTf		Adjusted R^2^ (%)
	β	*P*	β	*p*	β	*p*	β	*p*	
**CN**					
Volume	-0.259	***0.022***	0.006	0.971	-0.394	***0.014***	0.311	***0.005***	24.8
Volume % change	0.212	***0.043***	-0.280	0.054	-0.013	0.927	0.079	0.428	38.4
FDG	-0.021	0.910	0.228	0.468	-0.260	0.397	-0.058	0.764	3.80
FDG % change	0.368	***0.027***	-0.233	0.393	0.340	0.190	0.180	0.266	1.50
**MCI**									
Volume	0.094	0.379	-0.143	0.331	0.033	0.832	0.206	***0.016***	21.6
Volume % change	0.100	0.312	0.159	0.236	-0.260	0.076	0.115	0.135	38.9
FDG	0.160	0.284	0.281	0.226	-0.137	0.584	0.160	0.211	4.70
FDG % change	0.000	0.999	-0.056	0.774	-0.243	0.255	0.048	0.668	25.2
**AD**									
Volume	-0.026	0.856	0.047	0.816	-0.285	0.161	0.078	0.521	33.7
Volume % change	0.209	0.174	-0.245	0.270	-0.092	0.681	0.288	***0.036***	43.8
FDG	-0.027	0.895	-0.470	0.179	0.138	0.696	0.426	***0.019***	17.7
FDG % change	-0.072	0.802	0.214	0.642	-0.403	0.370	0.136	0.595	23.4


*Age and sex were included as covariates. The % change represents the longitudinal change in neuroimaging scores from baseline to the follow-up period for each respective patient. Since the follow-up time points were different between subjects, the follow-up time was also included as a covariate. Data is presented as mean ± standard deviation. The standardized coefficient (β) with adjusted R^2^ and *p*-values are stated, with significant *p*-values in bold italics.*

**Table 5:** Association of CSF Aβ, total tau (ttau), phosphorylated tau (ptau), and melanotransferrin (MTf) with cognitive scores in cognitively normal (CN), mild-cognitive impairment (MCI), or Alzheimer’s disease (AD) (Supplementary Figure 4).

	Aβ		Ttau		Ptau		MTf		Adjusted R^2^ (%)
	β	*P*	β	*p*	β	*p*	β	*p*	
**CN**									
MMSE	-0.198	0.121	0.188	0.296	-0.041	0.819	0.080	0.509	3.10
MMSE % change	0.364	***0.003***	-0.234	0.162	0.169	0.304	0.021	0.858	17.2
ADAS-Cog13	-0.242	***0.047***	0.016	0.925	-0.126	0.459	0.169	0.147	11.7
ADAS-Cog13 % change	-0.112	0.349	0.366	***0.030***	-0.078	0.636	-0.164	0.177	16.7
RAVLT	0.068	0.596	0.003	0.987	0.079	0.660	-0.131	0.283	1.90
RAVLT % change	0.284	***0.027***	-0.282	0.113	0.288	0.101	0.088	0.482	6.50
**MCI**									
MMSE	0.198	0.084	-0.038	0.813	0.013	0.077	-0.013	0.883	-0.400
MMSE % change	0.272	***0.006***	0.017	0.904	-0.102	0.490	0.141	0.072	26.2
ADAS-Cog13	-0.227	***0.041***	0.015	0.925	0.115	0.489	-0.172	***0.050***	7.00
ADAS-Cog13 % change	-0.140	0.177	-0.144	0.324	0.257	0.103	-0.049	0.550	17.8
RAVLT	0.079	0.481	-0.173	0.273	-0.007	0.969	0.138	0.123	2.90
RAVLT % change	0.174	0.094	0.103	0.480	-0.097	0.536	0.248	***0.003***	17.6
**AD**									
MMSE	0.211	0.211	0.048	0.851	-0.074	0.767	-0.081	0.579	-0.060
MMSE % change	0.143	0.366	-0.257	0.292	0.213	0.367	0.060	0.661	6.70
ADAS-Cog13	-0.083	0.620	0.306	0.230	-0.302	0.227	-0.181	0.216	-5.10
ADAS-Cog13 % change	-0.215	0.169	-0.011	0.964	0.066	0.775	0.091	0.500	9.80
RAVLT	0.236	0.146	-0.281	0.253	0.272	0.257	0.081	0.563	2.60
RAVLT % change	0.011	0.939	0.052	0.823	-0.061	0.788	-0.028	0.831	14.4


*Age and sex were included as covariates. The % change represents the longitudinal change in cognitive scores from baseline to the follow-up period for each respective patient. Since the follow-up time points were different between subjects, the follow-up time was also included as a covariate. Data is presented as mean ± standard deviation. The standardized coefficient (β) with adjusted R^2^ and *p*-values are stated, with significant *p*-values in bold italics.*

**FIGURE 1:**
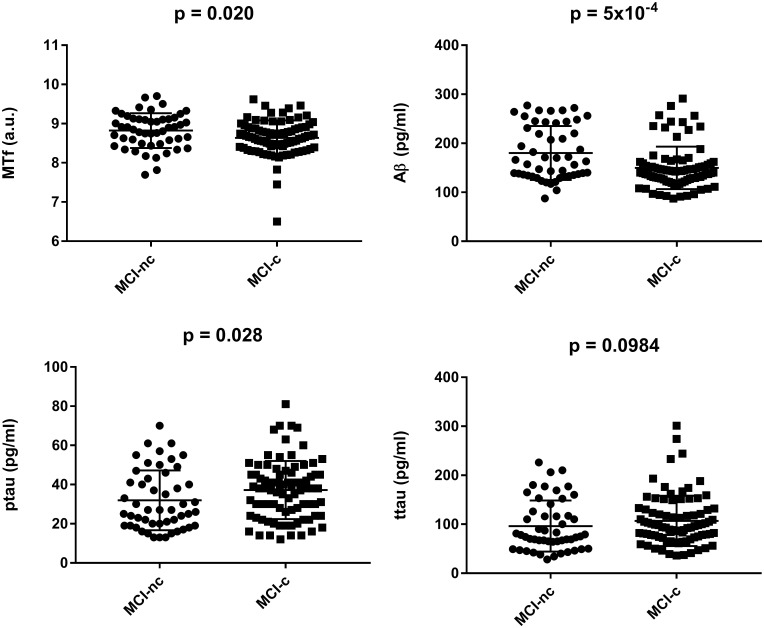
Baseline characteristics of mild cognitive impaired subjects stratified by conversion to Alzheimer’s Disease. Data are represented as mean ± standard deviation. The student’s *T*-test was used to test for differences in CSF melanotransferrin (MTf), Aβ, phosphorylated tau (ptau), and total tau (ttau) between non-converters (MCI-nc) and converters (MCI-c) with *p* < 0.05 being considered significant.

Interestingly, MCI subjects that converted to AD (MCI-c) demonstrated significantly lower levels of baseline CSF MTf compared to those that did not (MCI-nc; *p* = 0.020; Table 2 and Figure 1). MCI-c had significantly decreased Aβ (*p* = 5 × 10^-4^; Table 2 and Figure 1) and increased ptau compared to MCI-nc subjects (*p* = 0.028; Table 2 and Figure 1). Levels of ttau were similar between MCI-c and MCI-nc (*p* = 0.0984; Table 2 and Figure 1). Multiple regression modeling was also performed, including established AD biomarkers and CSF MTf according to the conversion status of MCI subjects (Table 6 and Supplementary Figures 5, 6), where CSF MTf was found to be positively correlated to percentage longitudinal change in RAVLT score in MCI-nc but not MCI-c (Table 6 and Supplementary Figure 6C).

**Table 6:** Association of CSF Aβ, total tau (ttau), phosphorylated tau (ptau), and melanotransferrin (MTf) with hippocampal neuroimaging measures and cognitive scores in MCI non-converters (MCI-nc) and converters (MCI-c) to Alzheimer’s Disease (Supplementary Figures 5, 6).

	Aβ		Ttau		Ptau		MTf		Adjusted R^2^ (%)
	β	*p*	β	*p*	β	*p*	β	*p*	
**Neuroimaging measures**					
MCI-nc									
Volume	0.202	0.201	-0.248	0.262	0.160	0.607	0.068	0.607	39.6
Volume % change	-0.075	0.651	1.052	***5.2 ×10*^-^*^5^***	-1.131	***6.3 ×10*^-^*^5^***	-0.043	0.763	33.1
FDG	0.153	0.450	-0.416	0.384	0.845	0.104	0.211	0.222	39.9
FDG % change	0.375	0.139	-0.235	0.675	0.041	0.946	0.028	0.888	11.0
MCI-c									
Volume	-0.106	0.382	0.008	0.963	-0.111	0.547	0.206	0.065	11.0
Volume % change	0.120	0.167	-0.058	0.640	-0.058	0.654	0.061	0.433	60.5
FDG	0.100	0.598	0.278	0.264	-0.204	0.435	-0.028	0.880	-0.081
FDG % change	-0.213	0.174	0.030	0.882	-0.316	0.152	-0.154	0.317	16.1
**Cognitive scores**					
MCI-nc									
MMSE	0.112	0.567	0.109	0.699	-0.098	0.750	0.097	0.553	-4.9
MMSE % change	0.618	**0.001**	-0.281	0.260	0.544	***0.049***	0.063	0.662	19.5
ADAS-Cog13	-0.326	0.083	0.175	0.515	-0.292	0.321	-0.155	0.323	5.5
ADAS-Cog13 % change	-0.166	0.379	-0.256	0.336	0.262	0.366	0.043	0.781	7.7
RAVLT	0.145	0.425	-0.181	0.491	0.259	0.367	0.107	0.483	9.6
RAVLT % change	0.199	0.279	-0.002	0.992	0.056	0.842	0.334	***0.029***	13.8
MCI-c									
MMSE	0.117	0.332	-0.111	0.527	0.150	0.418	-0.109	0.326	4.5
MMSE % change	0.144	0.182	0.194	0.221	-0.354	***0.036***	0.052	0.607	23.3
ADAS-Cog13	-0.103	0.389	-0.096	0.587	0.265	0.155	-0.148	0.185	5.6
ADAS-Cog13 % change	-0.010	0.927	-0.208	0.193	0.310	0.068	0.014	0.892	21.9
RAVLT	-0.052	0.671	-0.198	0.275	-0.031	0.871	0.074	0.516	-1.1
RAVLT % change	0.185	0.105	0.210	0.208	-0.237	0.182	0.109	0.303	14.5


*Age and sex were included as covariates. The % change represents the longitudinal change in neuroimaging/cognitive measures from baseline to the follow-up period for each respective patient. Since the follow-up time points were different between subjects, the follow-up time was also included as a covariate. Data is presented as mean ± standard deviation. The standardized coefficient (β) with adjusted R^2^ and *p*-values are stated, with significant *p*-values in bold italics.*

## Discussion

We demonstrate diminished levels of baseline CSF MTf are associated with lower hippocampal volumes in CN and MCI and worse cognitive scores in MCI subjects. Moreover, significantly lower levels of baseline CSF MTf were observed in MCI subjects converting to AD compared to non-converters, underscoring the possibility of CSF MTf to identify those individuals with increased susceptibility of converting to AD. In AD, lower CSF MTf levels was associated with a reduction in hippocampal volume over time and appear to reflect disease progression.

We found similar levels of CSF MTf in CN, MCI, and AD subjects. In contrast, a previous study demonstrated increased levels of CSF MTf in AD compared to individuals suffering from various neurodegenerative diseases (Kennard et al., 1996). However, our study cohort was a greater size, comprised mixed ethnicity and age-matched CN subjects, while the Kennard et al. study had only a Japanese cohort and the AD group older than the “control” group of individuals with non-AD neurodegenerative disease. With less genetic variability compared to our mixed ethnicity cohort, their results may not be representative of the general population. To the best of our knowledge, our study is the first report addressing the levels of CSF MTf levels in AD compared to CN subjects.

While elevated serum MTf have been found in AD (Kennard et al., 1996; Kim et al., 2001), others have observed this in early milder AD cases but no further increases in latter stages of AD (Kim et al., 2001). It is noted that serum MTf of varying glycosylated conformations were unchanged in another, but rather poor (see “Introduction”), study on AD subjects (Desrosiers et al., 2003). Desrosiers and co-workers used two-dimensional gel electrophoresis, whereas other, but validated studies, utilized sandwich fluorescent assay (Kennard et al., 1996), radioimmunoassay (Feldman et al., 2001), and dot-immunoblot assay (Kim et al., 2001) to measure serum MTf levels. The present study suffers from the limitation that plasma/serum levels of MTf were not available in the ADNI repository.

It is well documented that MCI subjects are at an increased risk of acquiring AD, and around 10–15% of these subjects convert to AD on a yearly basis (Risacher et al., 2009). By stratifying MCI individuals to MCI-c and MCI-nc, we found significantly diminished baseline CSF MTf levels in the former group. Indeed, lower CSF MTf were associated with greater cognitive deficits (ADAS-Cog13) in MCI and lower hippocampal volumes in both CN and MCI. While not associated with hippocampal volume, CSF MTf was associated with longitudinal hippocampal volume change in AD. MTf appears to have a role in mediating cellular iron uptake (Kennard et al., 1995), and hence likely to be involved in iron metabolism. Perturbed MTf expression may contribute to iron dysregulation and cellular iron accumulation, precipitating oxidative stress as iron is a potent source of free radicals, hastening AD pathogenesis. Greater iron content in the subcortical areas has been associated with poorer memory performance, lower general cognitive aptitude, mental retardation, and poorer cognitive and motor control in a healthy population (Sullivan et al., 2009; Penke et al., 2012; Rodrigue et al., 2013; Adamo et al., 2014; Daugherty and Raz, 2015). Furthermore, higher hippocampal iron has been correlated to smaller hippocampal volume, which in turn predicted poorer episodic memory (Rodrigue et al., 2013). Additionally, iron overload has been shown to accelerate cognitive impairment in human and transgenic mouse models of AD (Rodrigue et al., 2013; Becerril-Ortega et al., 2014). Interestingly, MCI subjects exhibited increased iron levels in the cortex and cerebellum (Smith et al., 2010). Another study reported an increase in the redox-active iron in the CSF of MCI but not AD cases, with levels correlating with the extent of cognitive impairment (Lavados et al., 2008). Consistent with these reports, we demonstrated a decrease in the baseline CSF MTf levels of MCI-c compared to MCI-nc, but no differences between CN, MCI, and AD groups, which suggests that iron dysregulation is an early event in AD pathogenesis (van Bergen et al., 2016).

There are two forms of MTf, one form is located on the cell surface via a GPI anchor on the plasma membrane, and the other is a soluble form that is secreted and found in the serum (Food et al., 1994; Desrosiers et al., 2003). Soluble MTf was originally thought to be derived from improper processing resulting in the protein evading the GPI-addition or endogenous (phosphatidylinositol-specific phospholipase D) cleavage of GPI-anchored MTf (McNagny et al., 1996). Moreover, soluble MTf was proposed to be generated from an alternatively spliced mRNA transcript lacking a GPI signal coding sequence (McNagny et al., 1996). However, more recently, Yang et al. (2004) conducted a detailed study in which deletion of the GPI pre-anchor sequence in human p97 led to a soluble form of MTf, and proposed more convincing mechanisms that could account for the soluble forms of GPI proteins. Apparently, three critical recognition sites are needed for the processing of GPI proteins in the endoplasmic reticulum: a transamidase, the residues to which the GPI anchor is linked and a carboxyl terminal signal peptide. Disruption in any of these could potentially alter MTf processing to result in soluble MTf, without a GPI anchor (Alemany et al., 1993; Maxwell et al., 1995; Yang et al., 2004). MTf in the blood has been known to undergo a high rate of transcytosis across the blood-brain-barrier (BBB) from the bloodstream to the brain (Demeule et al., 2002). We postulate that impaired transcytosis of MTf from the bloodstream into the brain may in part account for the decrease in CSF MTf levels in MCI-c and consistent with reports of increased serum MTf in AD (Kennard et al., 1996; Feldman et al., 2001; Kim et al., 2001). It is important to mention that MTf is not exclusively located in the brain but is also found in the liver and intestinal epithelial cells (Sciot et al., 1989; Alemany et al., 1993).

The low-density lipoprotein receptor (LRP) has been identified as a receptor for MTf and appears to actively transport MTf from the blood across the BBB into the brain (Demeule et al., 2002). Genetic studies strongly implicate the LRP gene locus in enhanced susceptibility to AD with APOE and Aβ being key LRP ligands (Kounnas et al., 1995; Kang et al., 1997). Furthermore, LRP levels are lower in AD, and of the two isoforms, LRP1 and LRP2, higher levels of the former have been associated with later onset of disease in AD patients, suggesting LRP1 may be protective against AD (Kang et al., 2000). At the BBB, LRP1 has been shown to be essential for the elimination of Aβ from the brain into the blood (Storck et al., 2016), with escalating Aβ levels in the brain associated with reduced LRP1 expression (Shibata et al., 2000). This reduction in LRP at the BBB may explain the lack of transcytosis of MTf from the blood into the brain and so, the low baseline CSF levels in MCI-c. However, it has been hypothesized that the brain capillary endothelial cells themselves produce MTf and that there is transcytosis in both directions at the BBB (Rothenberger et al., 1996). Thus, further investigations are needed to understand MTf import and export through the BBB and possible interactions with Aβ transport in aging and AD.

Neuroimaging and post-mortem studies have implicated BBB dysfunction as an early and common occurrence in AD, characterized by microbleeds, impaired glucose transport, disrupted functioning of P-glycoprotein 1, perivascular deposits of blood-derived proteins, cellular infiltration and degeneration of endothelial cells (Sweeney et al., 2018). Since the function of the BBB is to strictly regulate the blood-to-brain and brain-to-blood transport of solutes, MTf may simply leak through the disrupted BBB into the general circulation, leading to diminished levels of CSF MTf in the MCI-c at baseline. As the BBB is also known to be disrupted in AD, CSF MTf will also be expected to be reduced in AD, but this was not observed.

Additionally, the lower MTf levels in the CSF of MCI-c may, in part, arise from increased export of MTf from the CSF into the blood. LRP1 has also been detected at the choroid plexus (Pascale et al., 2011; Spuch et al., 2012) and suggests that MTf may be exported from the CSF to the blood via this route. Aβ (1–40) has been shown to be actively eliminated from the CSF, thought to be partly via LRP1 at the choroid plexus (Fujiyoshi et al., 2011). We propose that the lower CSF MTf in MCI-c in our study may result from its increased export from the CSF to the blood due to concomitant attenuated competition with the diminishing CSF Aβ levels arising from impaired Aβ clearance from the brain parenchyma. Future experiments are required to perform detailed investigations of whether other LRP substrates can influence the transcytosis of MTf, and the mechanisms governing MTf transport from the CSF to the blood and vice versa.

If CSF MTf is determined by Aβ clearance from the parenchyma into the CSF, this would imply that CSF MTf would be reduced in AD as levels of CSF Aβ are significantly reduced. A reduction in CSF MTf in AD was not observed in our study, but this may be explained by the increased production of MTf in the brain parenchyma from a subset of reactive microglia associated with amyloid plaques (Jefferies et al., 1996; Yamada et al., 1999). The subset of reactive (dysregulated) microglia surrounding plaques appear to be an exclusive hallmark of AD pathology and not observed in other neurodegenerative diseases (Jefferies et al., 1996; Rothenberger et al., 1996; Yamada et al., 1999). Whatever the mechanism(s) for reduced CSF MTf levels in MCI-c (described above), they are likely to be operational in AD as well and ought to lead to reduced CSF MTf in AD, we propose this is not observed as levels are maintained by MTf production from these reactive microglia surrounding plaques. The microglia appear to be laden with iron, as evident by high expression of the iron storage protein, ferritin (McGeer et al., 1987; Grundke-Iqbal et al., 1990; Food et al., 1994). We speculate that increased iron uptake by the reactive microglia via expression of GPI-anchored MTf leads to cellular iron accumulation, further exacerbating microglial dysfunction. Chinese hamster ovary (CHO) cells defective in the transferrin receptor but transfected to express MTf, showed doubling of iron intake. Iron-associated microglial-driven neuroinflammation may be a significant driver behind neuronal and synaptic destruction, working synergistically with Aβ (Gallagher et al., 2012; McGeer and McGeer, 2013). Determining the source of CSF MTf may aid determination of the defect that is contributing to AD pathogenesis.

Based on our results, CSF MTf levels appears to be significantly decreased in MCI-c compared to MCI-nc at baseline. However, no significant changes were found in MTf levels at the AD stage. MTf may be involved in iron metabolism, iron dyshomeostasis may be an early event in disease pathogenesis. Indeed, redox-active CSF iron levels were shown to be increased from normal to MCI subjects, while in AD, there was an abrupt decrease in iron levels close to zero (Lavados et al., 2008). Through histochemical iron analysis, increased brain iron content was reported in MCI and preclinical cases of AD (Smith et al., 2010). It is likely that perturbations in baseline MTf levels and resultant dysfunction at the stage of MCI, may be a significant and early contribution to the disease process prior to acquiring AD.

## Conclusion

In conclusion, we demonstrate that baseline CSF MTf levels are significantly decreased in MCI-c compared to MCI-nc. However, our results remain to be validated in an independent cohort. Future directions would be required to elucidate the role of MTf in the context of AD, especially determining the source of MTf in the CSF and how brain MTf is regulated by cellular barriers, Aβ and activated microglial cells in human and transgenic AD models, requiring measurements in paired blood-CSF samples. Nevertheless, our study implies that baseline CSF MTf levels may be a useful marker to identify individuals with increased risk of conversion to AD, although much development still needs to be undertaken to ensure robust assay reproducibility across multiple clinical laboratories.

## Data Availability

Publicly available datasets were analyzed in this study. This data can be found here: http://adni.loni.usc.edu/.

## Author Contributions

AA and P-WS contributed the concept and design of the study, wrote the first draft of the manuscript, and revised and approved the submitted version. AA performed the statistical analysis, aided by JA, MA, and CH, with interpretation performed by AA and P-WS.

## Conflict of Interest Statement

The authors declare that the research was conducted in the absence of any commercial or financial relationships that could be construed as a potential conflict of interest.

## Footnotes

1 http://adni.loni.usc.edu/

2 http://www.adni-info.org/

3 ida.loni.usc.edu

4 http://www.adni-info.org/Scientists/doc/PET-Tech_Procedures_Manual_v9.5.pdf

5 http://adni.loni.usc.edu/methods/pet-analysis/pre-processing/

6 http://www.adni-info.org
