# Supplementary material for: Low Cerebrospinal Fluid Levels of Melanotransferrin Are Associated With Conversion of Mild Cognitively Impaired Subjects to Alzheimer’s Disease
Source: Front Neurosci. 2019 Mar 8;13:181. doi: 10.3389/fnins.2019.00181 (PMC6419538; doi:10.3389/fnins.2019.00181)

Low cerebrospinal fluid levels of melanotransferrin are associated with conversion of mild cognitively impaired subjects to Alzheimer's disease

Azhaar Ashraf[1], Jose Andres Alepuz Guillen[1], Manal Aljuhani[1], Chantal Hubens[1], Po-Wah So[1]*, for Alzheimer's Disease Neuroimaging Initiative

[1]Department of Neuroimaging, Institute of Psychiatry, Psychology and Neuroscience, King's College London, London, United Kingdom.

*Correspondence to:

Dr Po-Wah So,

King's College London,

Institute of Psychiatry, Psychology and Neuroscience,

Department of Neuroimaging,

Maurice Wohl Clinical Neuroscience Institute,

5, Cutcombe Road,

London. SE5 9RX

Email: po-wah.so@kcl.ac.uk.

Telephone: +44 (0)20 7848 5453

Table 1: The n per group of ADNI cohort with 10-year follow-up, based on diagnosis (cognitively normal (CN), mild cognitive impairment (MCI) and Alzheimer's disease (AD)) for neuroimaging measures and cognitive assessments.

Follow-up (years)	0	1	2	3	4	5	6	7	8	9	10
**Volume**											
**CN**	3	6	8	19	9	6	22	1	5	7	0
**MCI**	17	9	35	27	11	11	15	4	4	2	0
**AD**	17	16	33	0	0	0	0	0	0	0	0
**FDG**											
**CN**	26	1	3	13	4	4	18	15	2	1	3
**MCI**	53	2	20	24	6	4	15	9	1	0	1
**AD**	28	7	21	0	0	0	0	0	0	0	0
**MMSE**											
**CN**	1	1	6	17	6	1	9	4	11	12	18
**MCI**	0	4	30	39	14	8	10	7	4	12	7
**AD**	7	8	51	0	0	0	0	0	0	0	0
**ADAS-Cog13**											
**CN**	2	1	7	16	5	1	9	4	9	14	18
**MCI**	0	4	30	39	13	8	10	7	7	10	7
**AD**	5	12	49	0	0	0	0	0	0	0	0
**RAVLT**											
**CN**	1	1	7	16	6	1	9	5	9	13	18
**MCI**	0	14	23	36	16	6	9	11	3	10	7
**AD**	6	11	49	0	0	0	0	0	0	0	0

Figure 1: Modelling the association of (A) hippocampal volume and (B) % change; and (C) hippocampal glucose/FDG metabolism (D) and % change, with (i) CSF Aβ, (ii) total tau (ttau), (iii) phosphorylated tau (ptau) and (iv) melanotransferrin (MTf) in the total cohort. Age, sex and diagnosis were included as covariates. The % change represents the longitudinal change in neuroimaging measures from baseline to the follow-up period for each patient. Since follow-up time points were different between subjects, follow-up time was also included as a covariate. The standardized coefficient (β) values are stated with significance levels, $p < 0.05$*, $p < 0.01$** and $p < 0.005$***.

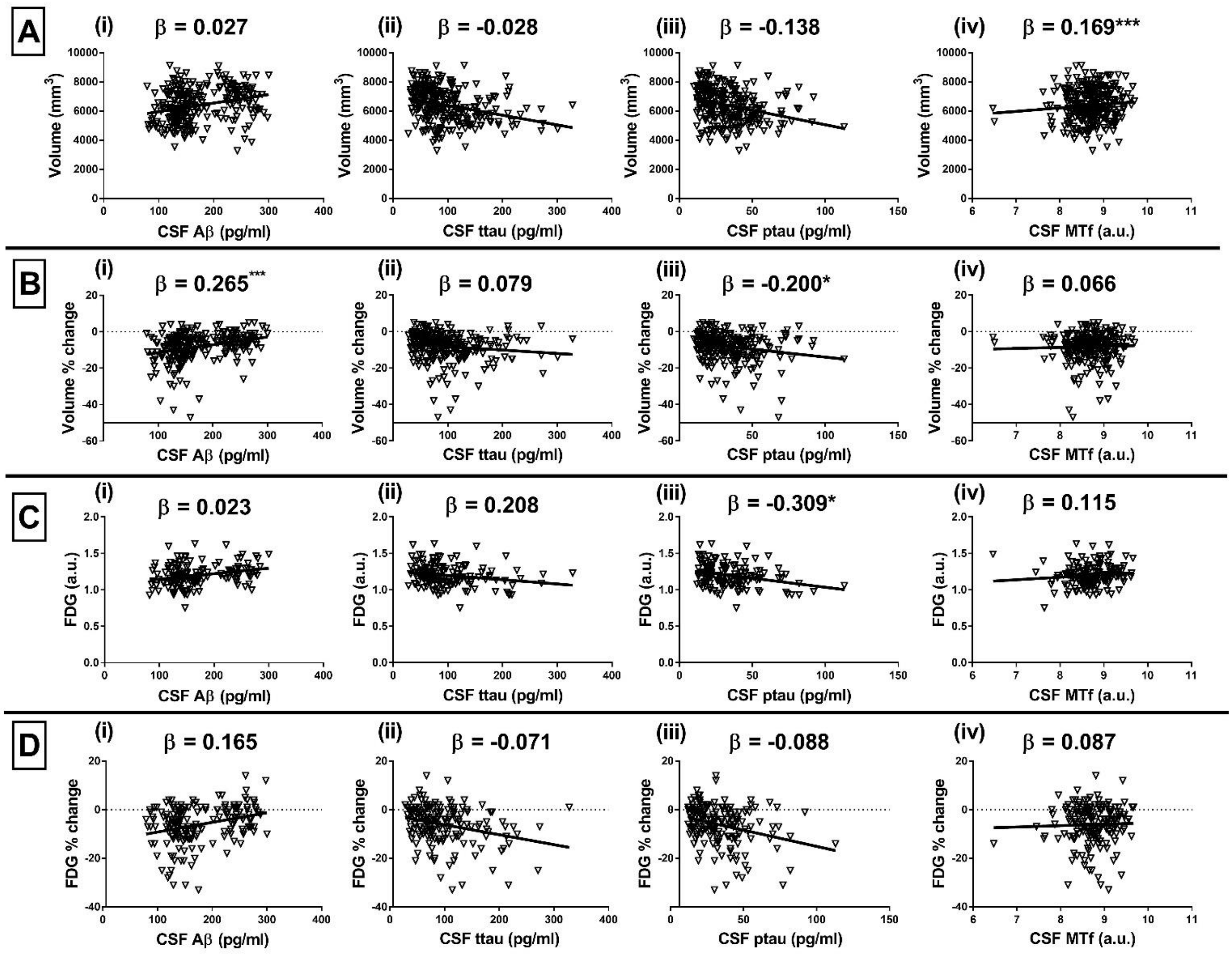

Figure 2: Modelling the association of (A) MMSE and (B) % change; (C) ADAS-Cog13 scores and (D) % change; (E) RAVLT scores and (F) % change, with (i) CSF Aβ, (ii) total tau (ttau), (iii) phosphorylated tau (ptau) and (iv) melanotransferrin (MTf) in the total cohort. Age, sex and diagnosis were included as covariates. The % change represents the longitudinal change in cognitive measures from baseline to the follow-up period for each patient. Since the follow-up time points were different between subjects, follow-up time was also included as a covariate. The standardized coefficient (β) values are stated with significance, $p < 0.05$*, $p < 0.01$** and $p < 0.005$***.

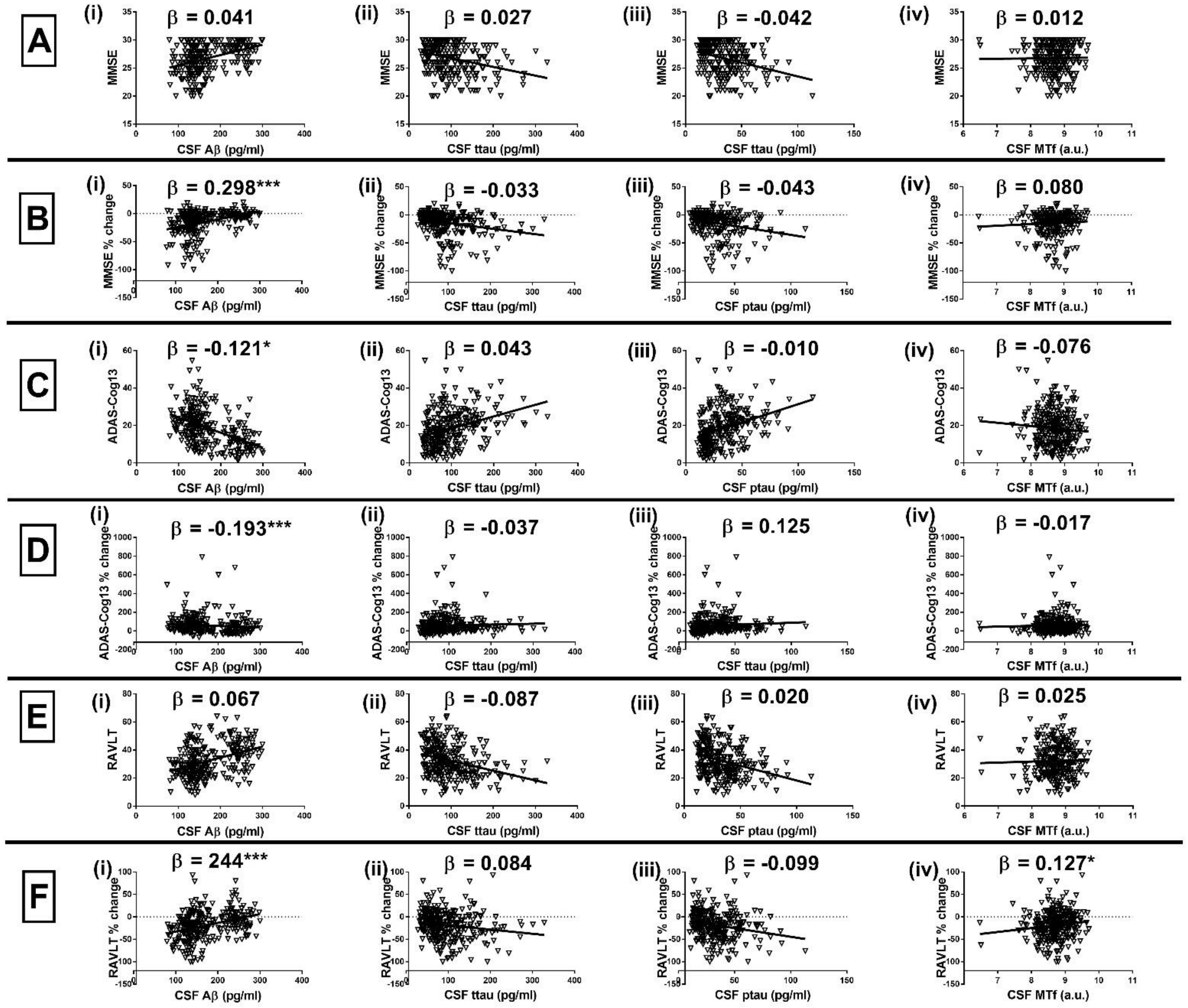

Figure 3A: Association of (A) hippocampal baseline volume and (B) % change, with (i) CSF Aβ, (ii) total tau (ttau), (iii) phosphorylated tau (ptau) and (iv) melanotransferrin (MTf) in cognitively normal (CN, top panel), mild cognitive impairment (MCI, middle panel) or Alzheimer's disease (AD, bottom panel). Age and sex were included as covariates. The % change represents the longitudinal change in MRI volume scores from baseline to the follow-up period for each patient. Since the follow-up time points were different between subjects, follow-up time was also included as a covariate. The standardized coefficient (β) values are stated with significance levels, p < 0.05*, p < 0.01** and p < 0.005***.

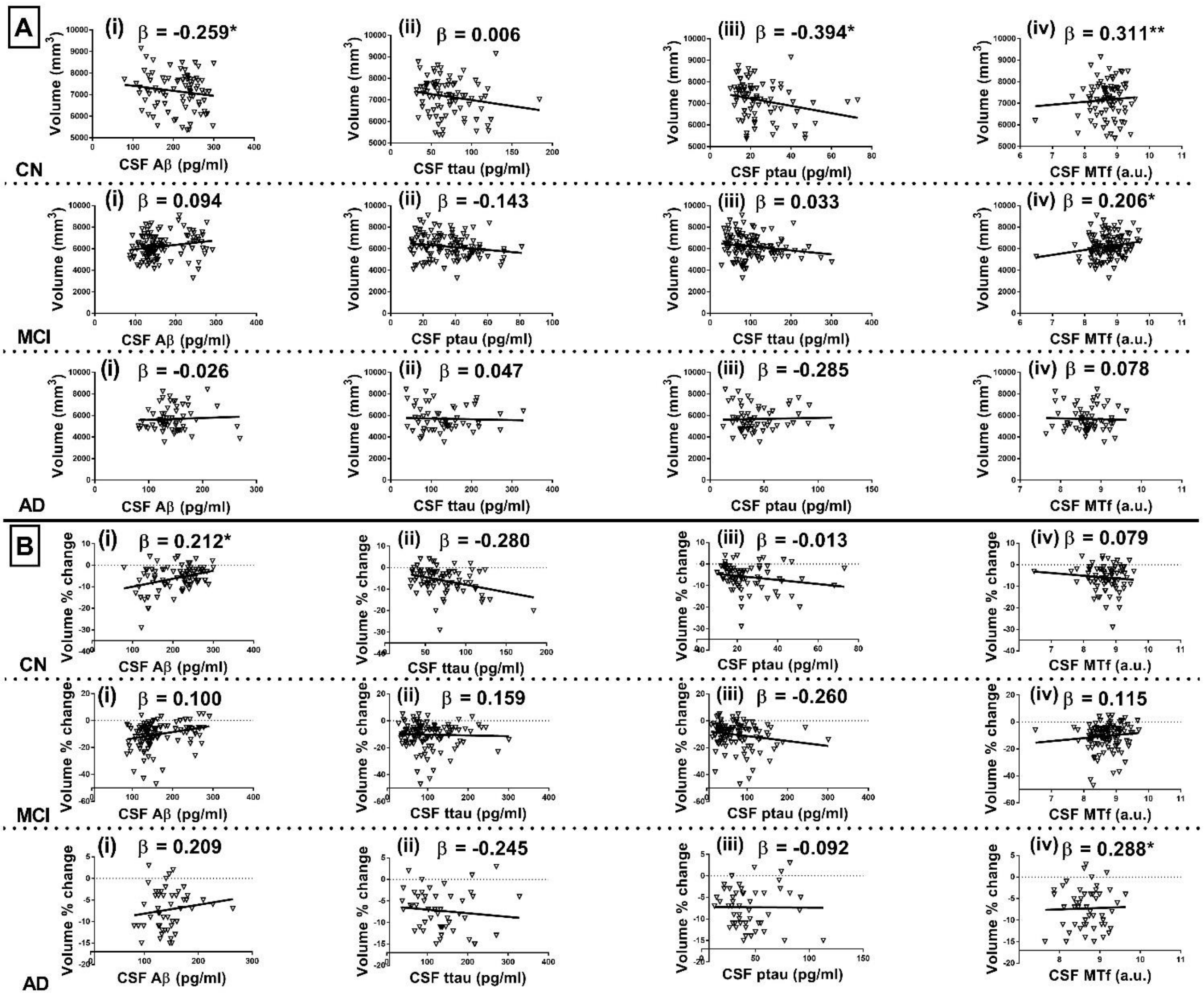

Figure 3B: Association of (A) hippocampal baseline glucose/FDG metabolism and (B) % change, with (i) CSF Aβ, (ii) total tau (ttau), (iii) phosphorylated tau (ptau) and (iv) melanotransferrin (MTf) in cognitively normal (CN, top panel), mild cognitive impairment (MCI, middle panel) or Alzheimer's disease (AD, bottom panel). Age and sex were included as covariates. The % change represents the longitudinal change in glucose/FDG scores from baseline to the follow-up period for each patient. Since the follow-up time points were different between subjects, follow-up time was also included as a covariate. The standardized coefficient (β) values are stated with significance levels, $p < 0.05$*, $p < 0.01$** and $p < 0.005$***.

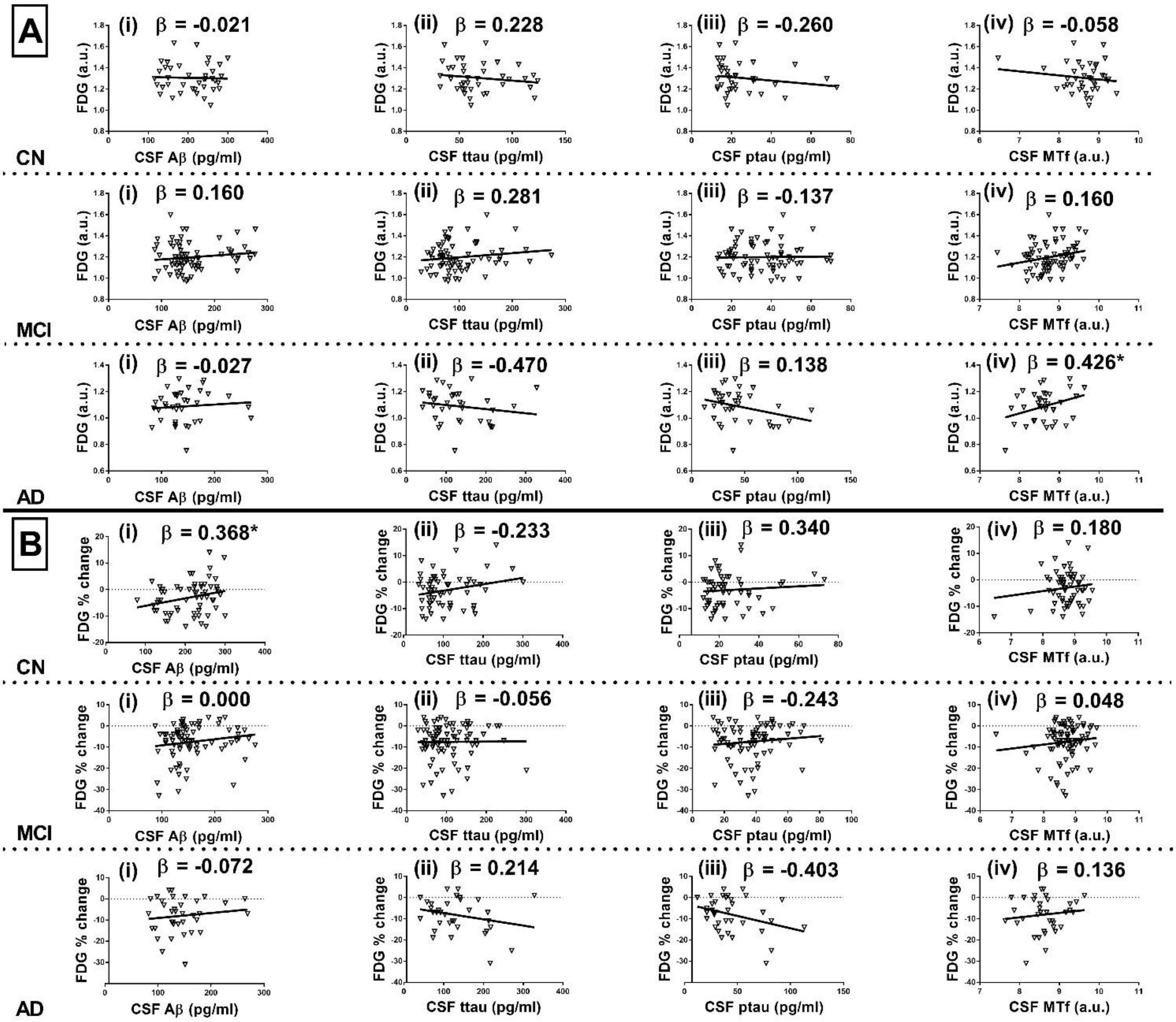

Figure 4A: Association of (A) baseline MMSE and (B) % longitudinal MMSE change, with (i) CSF Aβ, (ii) total tau (ttau), (iii) phosphorylated tau (ptau) and (iv) melanotransferrin (MTf) in cognitively normal (CN, top panel), mild-cognitive impairment (MCI, middle panel) or Alzheimer's disease (AD, bottom panel). Age and sex were included as covariates. The % change represents the longitudinal change in MMSE scores from baseline to the follow-up period for each patient. Since the follow-up time points were different between subjects, follow-up time was also included as a covariate. The standardized coefficient (β) values are stated with significance levels, p<0.05*, p< 0.01** and p<0.005***.

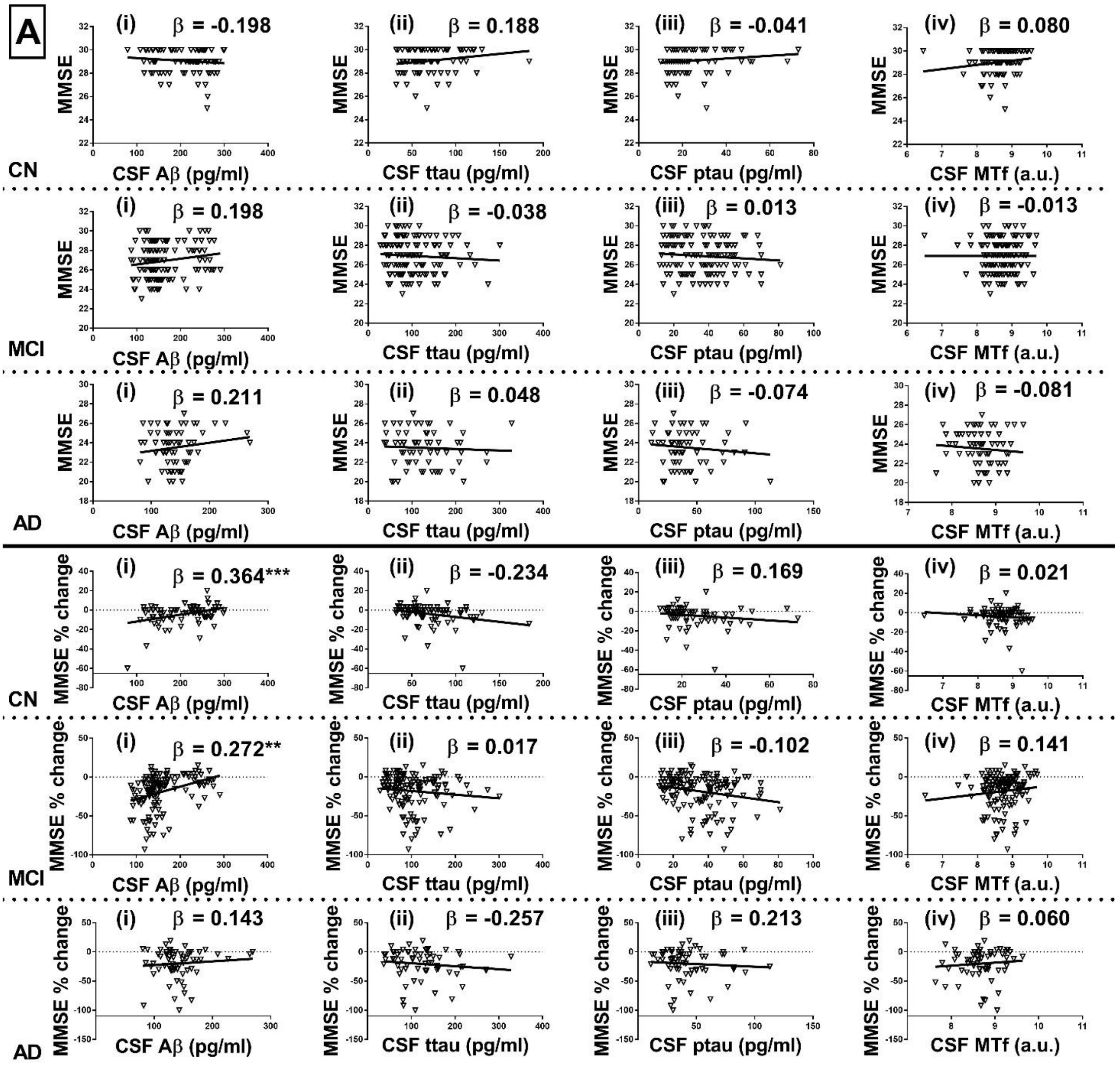

Figure 4B: Association of (A) baseline ADAS-Cog13 scores and (B) % longitudinal change in ADAS-Cog13 scores, with (i) CSF Aβ, (ii) total tau (ttau), (iii) phosphorylated tau (ptau) and (iv) melanotransferrin (MTf) in cognitively normal (CN, top panel), mild-cognitive impairment (MCI, middle panel) or Alzheimer's disease (AD, bottom panel). Age and sex were included as covariates. The % change represents the longitudinal change in ADAS-Cog13 scores from baseline to the follow-up period for each patient. Since the follow-up time points were different between subjects, follow-up time was also included as a covariate. The standardized coefficient (β) values are stated with significance levels, $p<0.05^{*}$, $p< 0.01^{**}$ and $p<0.005^{***}$.

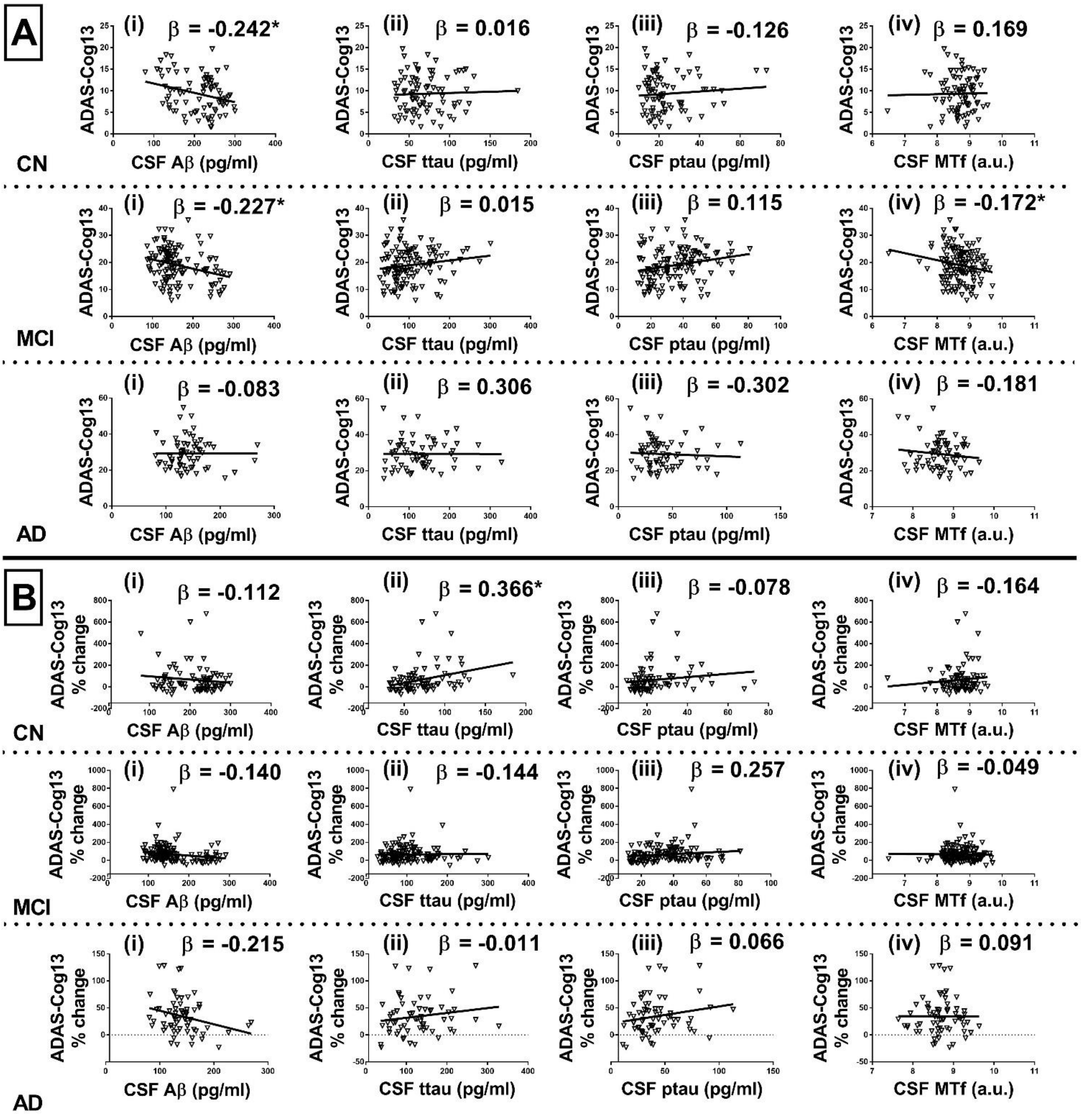

Figure 4C: Association of (A) baseline RAVLT scores and (B) % longitudinal RAVLT change, with (i) CSF Aβ, (ii) total tau (ttau), (iii) phosphorylated tau (ptau) and (iv) melanotransferrin (MTf) in cognitively normal (CN, top panel), mild-cognitive impairment (MCI, middle panel) or Alzheimer's disease (AD, bottom panel). Age and sex were included as covariates. The % change represents the longitudinal change in RAVLT scores from baseline to the follow-up period for each patient. Since the follow-up time points were different between subjects, follow-up time was also included as a covariate. The standardized coefficient (β) values are stated with significance levels, p<0.05*, p< 0.01** and p<0.005***.

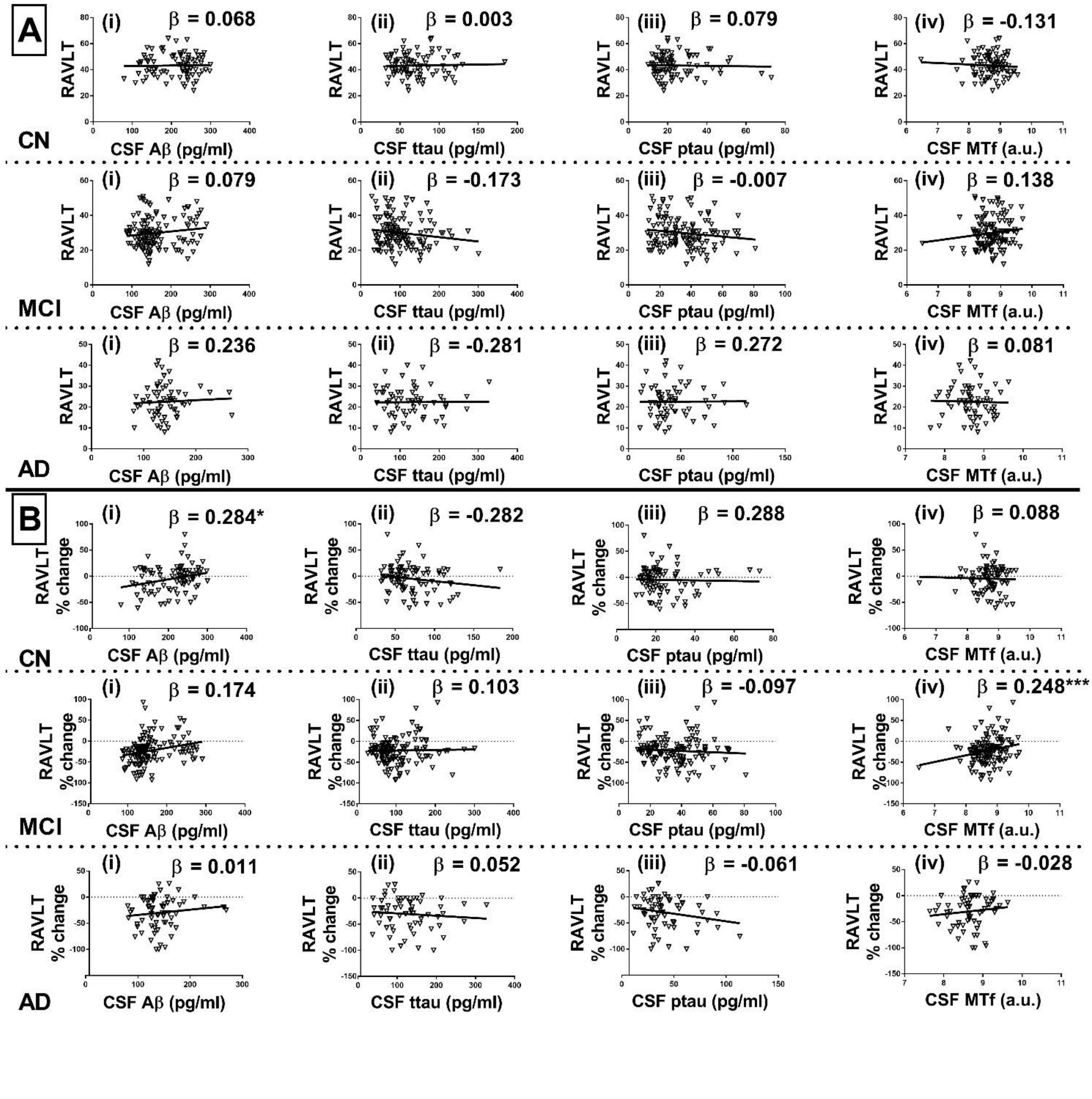

Figure 5A: Association of hippocampal (A) baseline volume and (B) % longitudinal change (B, with (i) CSF Aβ, (ii) total tau (ttau), (iii) phosphorylated tau (ptau) and (iv) melanotransferrin (MTf) in mild cognitive impaired (MCI) non-converters (MCI-nc, top panel) and converters to Alzheimer's Disease (MCI-c, bottom panel). Age and sex were included as covariates. The % change represents the longitudinal change in MRI volumes from baseline to the follow-up period for each patient. Since the follow-up time points were different between subjects, the follow-up time was also included as a covariate. The standardized coefficient (β) values are stated with significance, p<0.05*, p<0.01** and p<0.005***.

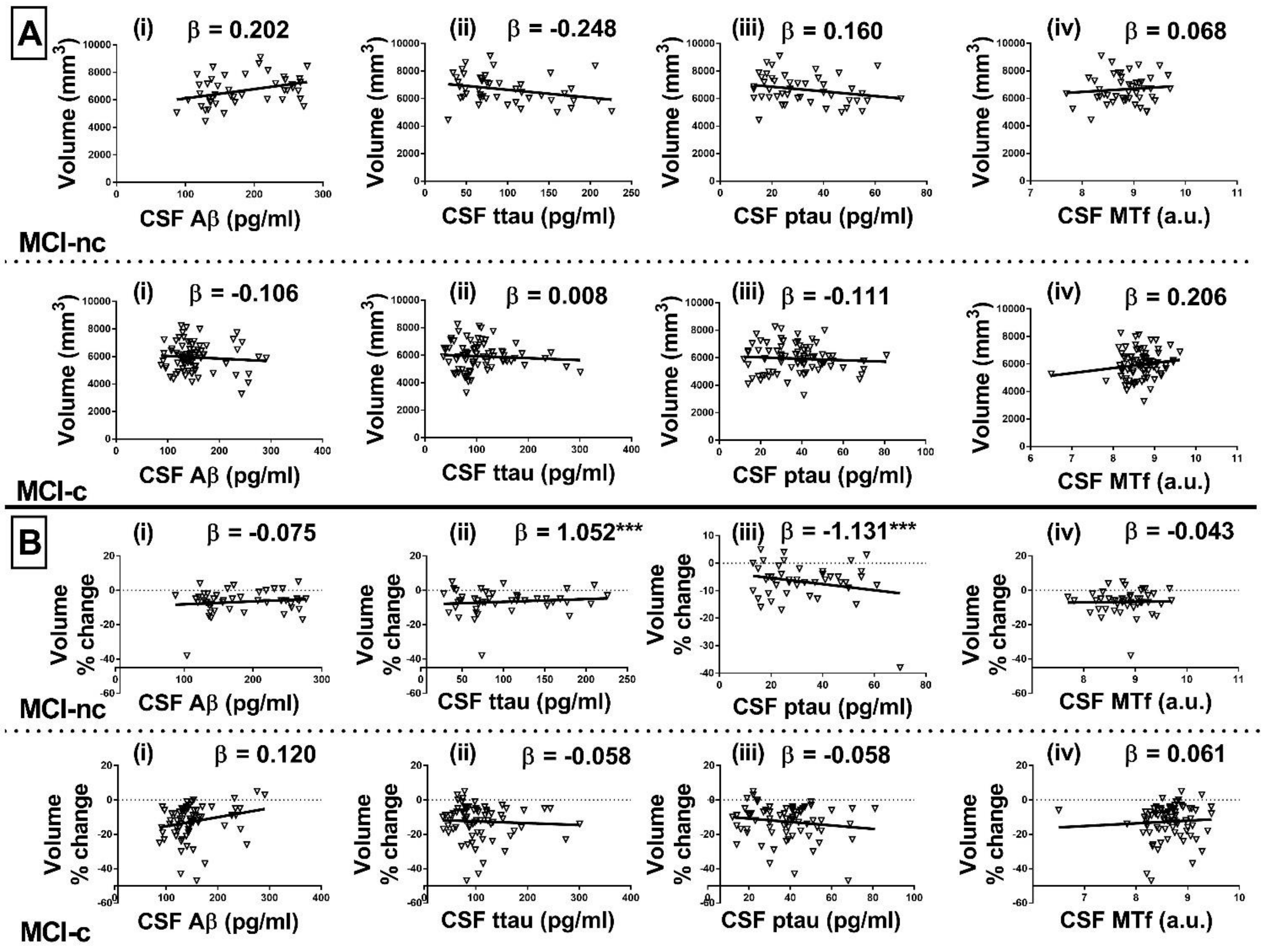

Figure 5B: Association of (A) baseline glucose/FDG metabolism and (B) % longitudinal change, with (i) CSF Aβ, (ii) total tau (ttau), (iii) phosphorylated tau (ptau) and (iv) melanotransferrin (MTf) in mild cognitive impaired (MCI) non-converters (MCI-nc, top panel) and converters to Alzheimer's Disease (MCI-c, bottom panel). Age and sex were included as covariates. The % change represents the longitudinal change in glucose/FDG metabolism from baseline to the follow-up period for each patient. Since the follow-up time points were different between subjects, the follow-up time was also included as a covariate. The standardized coefficient (β) values are stated with significance, $p<0.05$*, $p<0.01$** and $p<0.005$***.

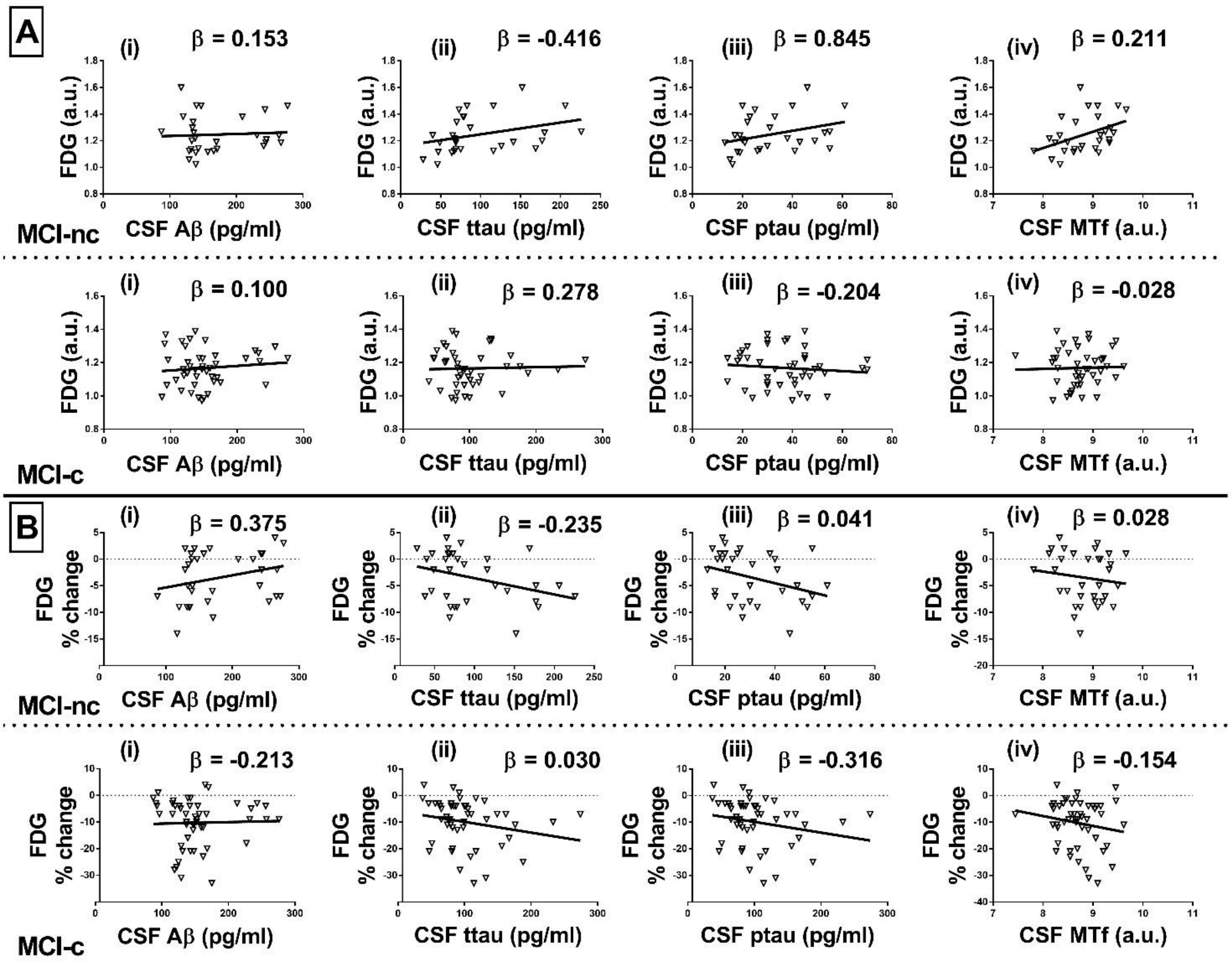

Figure 6A: Association of (A) baseline MMSE and (B) % longitudinal change, with (i) CSF Aβ, (ii) total tau (ttau), (iii) phosphorylated tau (ptau) and (iv) melanotransferrin (MTf) in mild cognitive impaired (MCI) non-converters (MCI-nc, top panel) and converters to Alzheimer's Disease (MCI-c, bottom panel). Age and sex were included as covariates. The % change represents the longitudinal change in MMSE scores from baseline to the follow-up period for each patient. Since the follow-up time points were different between subjects, the follow-up time was also included as a covariate. The standardized coefficient (β) values are stated with significance, $p<0.05$*, $p<0.01$** and $p<0.005$***.

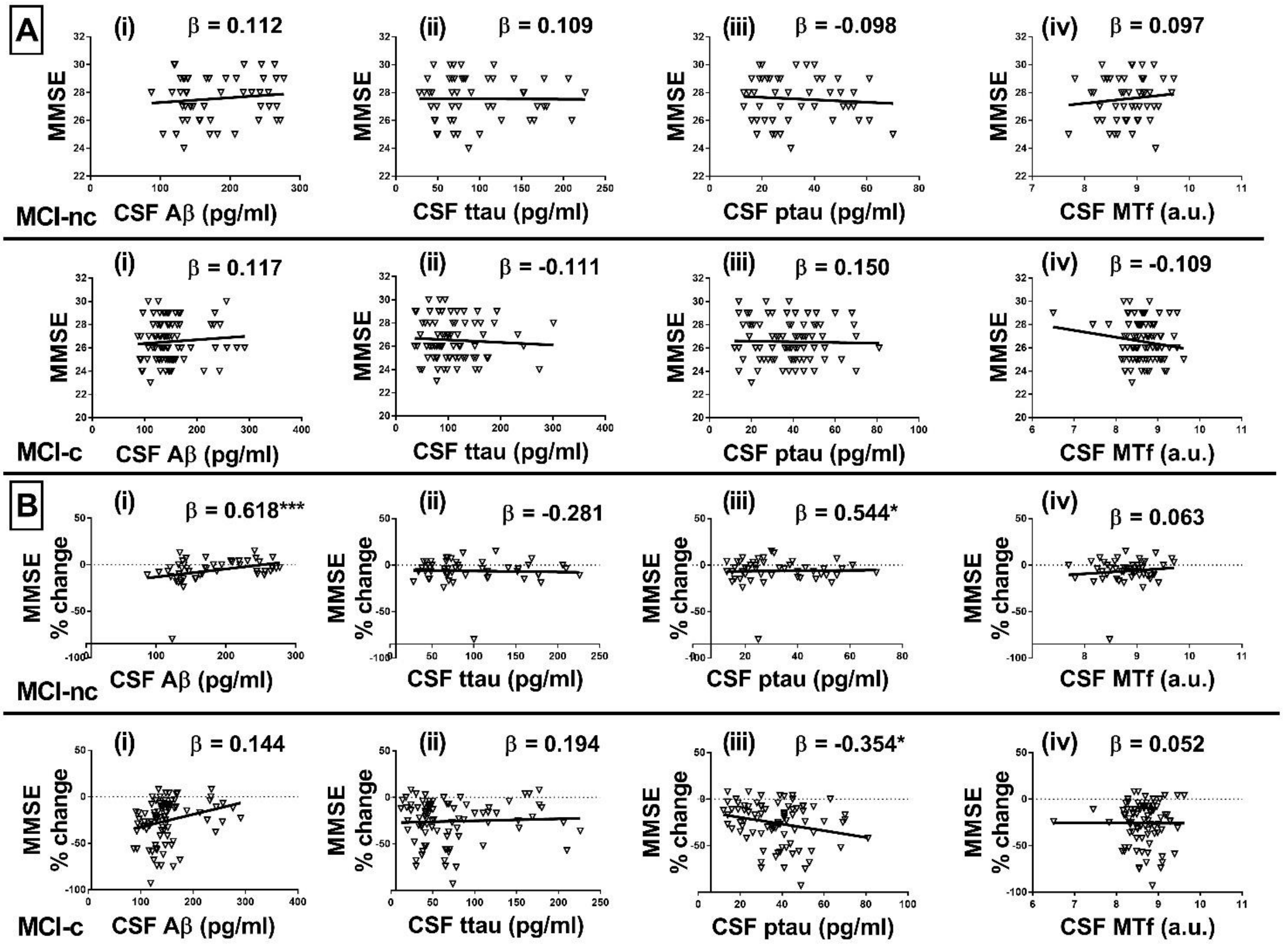

Figure 6B: Association (A) baseline ADAS-Cog13 scores and (B) % longitudinal change, with (i) CSF Aβ, (ii) total tau (ttau), (iii) phosphorylated tau (ptau) and (iv) melanotransferrin (MTf) in mild cognitive impaired (MCI) non-converters (MCI-nc, top panel) and converters to Alzheimer's Disease (MCI-c, bottom panel). Age and sex were included as covariates. The % change represents the longitudinal change in ADAS-Cog13 scores from baseline to the follow-up period for each patient. Since the follow-up time points were different between subjects, the follow-up time was also included as a covariate. The standardized coefficient (β) values are stated with significance, p<0.05*, p<0.01** and p<0.005***.

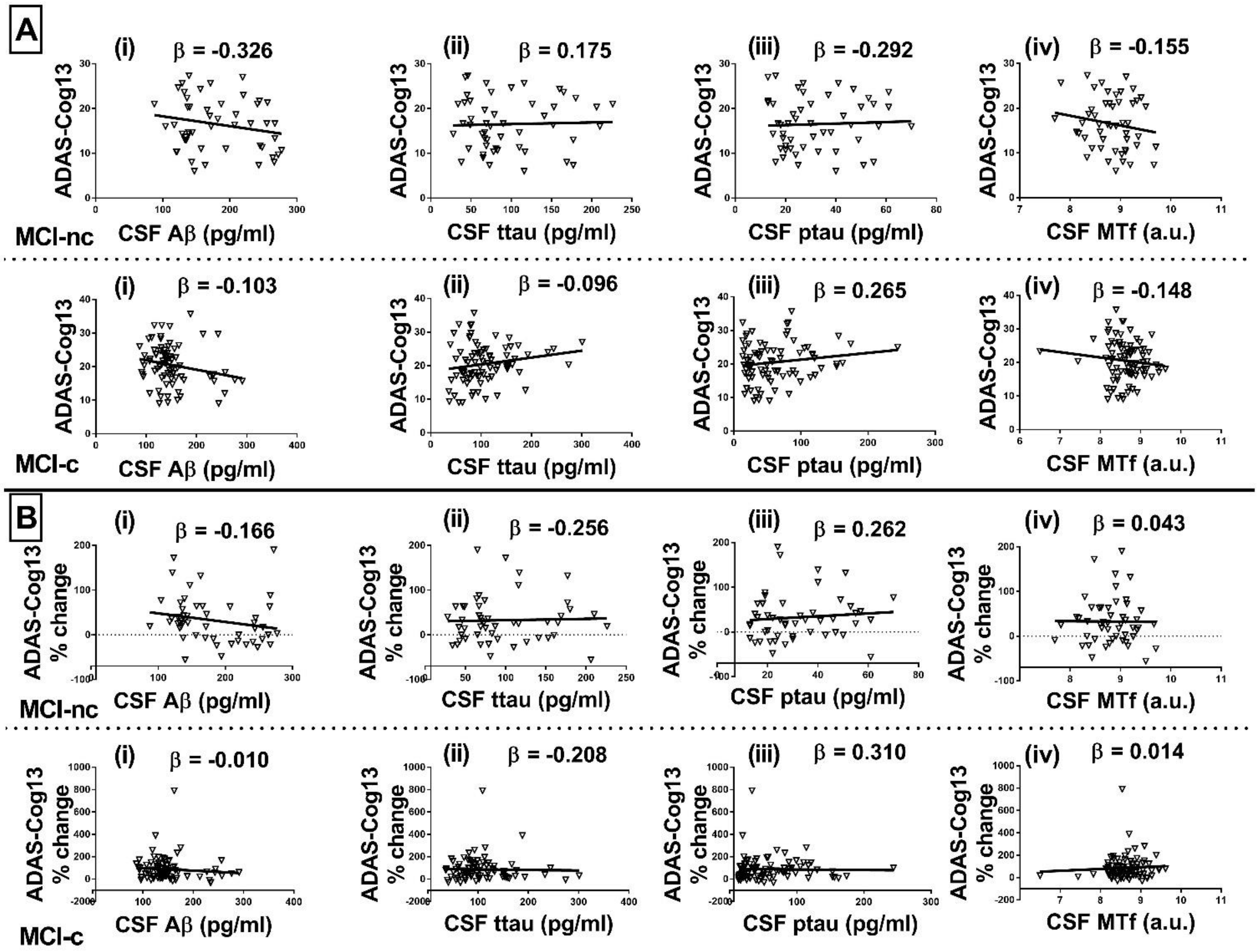

Figure 6C: Association of (A) RAVLT and (B) % longitudinal change, with (i) CSF Aβ, (ii) total tau (ttau), (iii) phosphorylated tau (ptau) and (iv) melanotransferrin (MTf) in mild cognitive impaired (MCI) non-converters (MCI-nc, top panel) and converters to Alzheimer's Disease (MCI-c, bottom panel). Age and sex were included as covariates. The % change represents the longitudinal change in RAVLT scores from baseline to the follow-up period for each patient. Since the follow-up time points were different between subjects, the follow-up time was also included as a covariate. The standardized coefficient (β) values are stated with significance, $p<0.05$*, $p<0.01$** and $p<0.005$***.

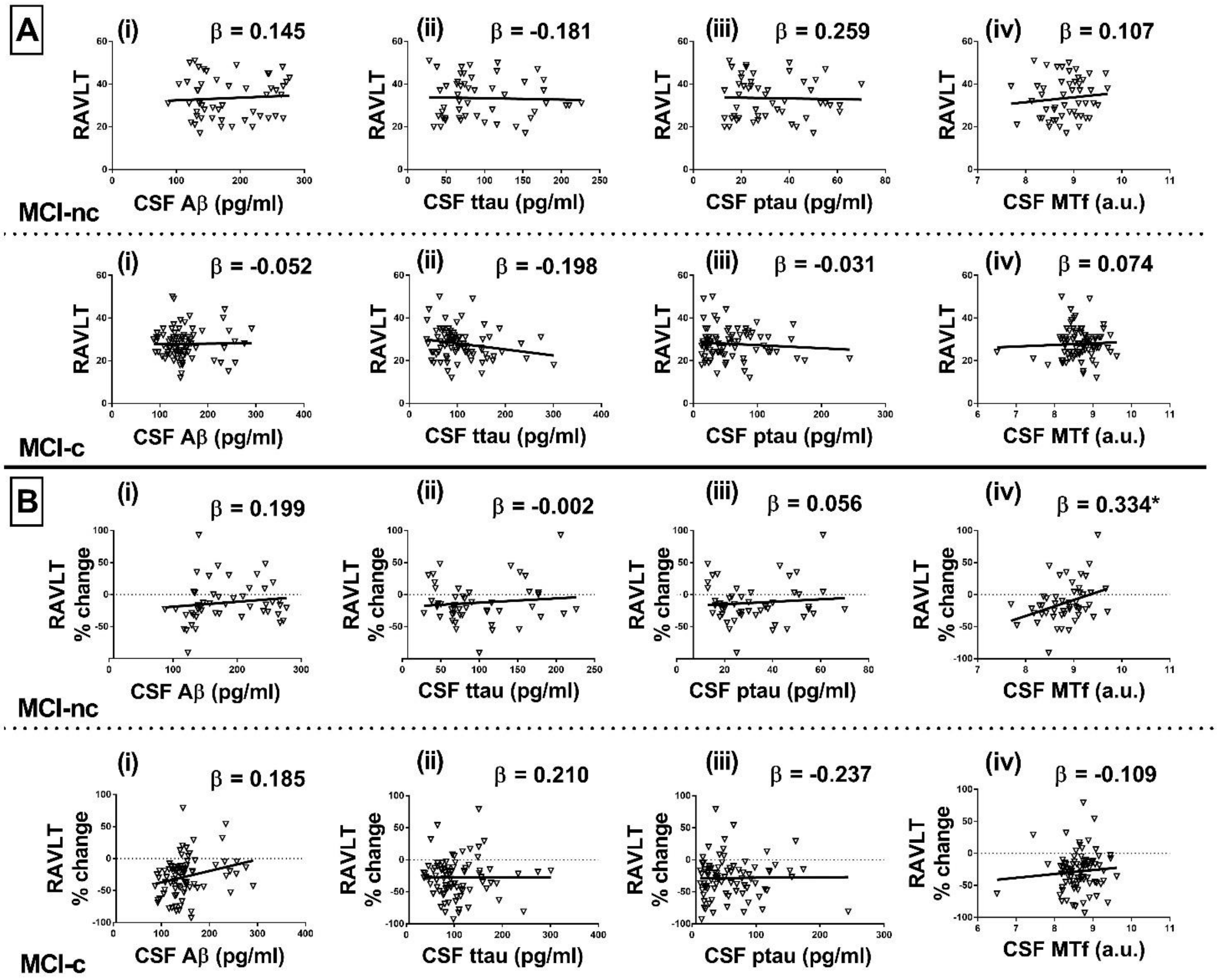

Supplement: Supplementary file 1 [file Data_Sheet_1.PDF]
